# Supplementary material for: WordCommentsAnalyzer: A windows software tool for qualitative research
Source: F1000Res. 2018 Sep 4;7:536. Originally published 2018 May 3. [Version 2] doi: 10.12688/f1000research.14819.2 (PMC6137411; doi:10.12688/f1000research.14819.2)
Supplement: Supplementary file 3 [file f1000research-7-17614-s0002.tgz › b2a79496-4ada-47c4-9682-a99ce867836d.zip!/Eight Iranian Health Journals/Journal of Health System Research _28.docx]

© Journal of Health System Research . This work is licensed under CC BY-NC 4.0 (https://creativecommons.org/licenses/by-nc/4.0/).

Volume & Issue: دوره 9، شماره 4؛ 1392

Title: اسیدهای چرب امگا 3 و درمان اختلالات روحی

http://hsr.mui.ac.ir/index.php/jhsr/article/view/777

مقدمه: امروزه تعداد زيادي از مطالعات مشاهده‌اي و اپيدميولوژيكي پيشنهاد مي‌كنند كه اختلالات رفتاري با كاهش دريافت اسيدهاي چرب امگا 3 در ارتباط مي‌باشند. اين شواهد باعث انجام مطالعات كارآزمايي باليني به منظور بررسي اثر اين اسيدهاي چرب در اختلالات رفتاري شدند. در مطالعه مروري حاضر، كليه كارآزمايي‌هاي باليني كه از سال 2009 به بعد به منظور بررسي اثر اسيدهاي چرب امگا 3 روي اختلالات رفتاري منتشر شده بودند، مورد بررسي قرار گرفتند. روش‌ها: در بسياري از مطالعات حجم نمونه خيلي كوچك بود، ولي به دليل طراحي بسيار عالي، در این مقاله بررسی شدند. اسيدهاي چرب امگا 3 هم در كودكان و هم در بزرگسالان خيلي خوب تحمل مي‌شد و تنها عوارض جانبي گزارش شده، ناراحتي‌های جزیي گوارشي بود كه در خيلي از بيماران ديده شد. در مورد اسكيزوفرني، خيلي از شواهد نشان دهنده مؤثر بودن اسيد چرب امگا 3 در درمان بيماري است. در مورد بيش‌فعالي- نقص توجه نتايج نشان دهنده تأثير جزیي اسيدهاي چرب امگا 3 در درمان اين بيماري است، ولي تأثيرات سودمند امگا 3 در مدرسه بيشتر از محيط خانواده گزارش شد. در مورد افسردگي ممكن است اسيد چرب ايكوزاپنتوئنوئیک اسيد مؤثرتر از دوكزاهگزا انوئيك اسيد باشد، ولي به علت عوامل مخدوش كننده فراوان نمي‌توان به طور قطعي اظهار نظر كرد. نتيجه‌گيري: با وجود اين‌كه نمي‌توان به طور قطعي اسيدهاي چرب امگا 3 را به عنوان درمان اختلالات روان توصيه كرد، اما شواهد در دسترس كه توجيه كننده انجام مطالعات باليني بيشتري مي‌باشند، بسيار قوي هستند.

Title: بررسی وضعیت تضاد شغلی و تضاد خانواده و ارتباط آن با کیفیت زندگی پرستاران شاغل در بالین

http://hsr.mui.ac.ir/index.php/jhsr/article/view/775

مقدمه: پرستاران همواره با حجم کار زیاد و منابع انسانی محدود مواجه هستند و از طرف دیگر باید نقش خود را به عنوان عضو مؤثر در خانواده ایفا نمایند. با توجه به این‌که لازم است پرستاران در برقراری تعادل بین مسؤولیت‌های کاری و خانوادگی خود ایفای نقش نمایند، ممکن است این امر بر کیفیت زندگی آن‌ها تأثیرگذار باشد. مطالعه حاضر با هدف، ارزیابی وضعیت تضاد شغلی و تضاد خانواده با شغل در پرستاران شاغل در بیمارستان و ارتباط بین این عوامل با کیفیت زندگی آنان طراحی و اجرا گردید. روش‌ها: این پژوهش یک مطالعه مقطعي بود که در آن 60 پرستار شاغل در سه بیمارستان آموزشی شهر تهران با نمونه‌گیری تصادفی ساده انتخاب شدند. ابزار جمع‌آوری داده‌ها شامل چهار قسمت اطلاعات دموگرافیک، پرسش‌نامه‌های تضاد شغل با خانواده و تضاد خانواده با شغل Carlson و پرسش‌نامه کیفیت زندگی (SF-36) بود. برای تجزیه و تحلیل داده‌ها از روش آمار توصیفی و همبستگی استفاده گردید و سطح معنی‌داری در این مطالعه 05/0 در نظر گرفته شد. یافته‌ها: میزان تضاد شغل با خانواده در پرستاران به مراتب بیشتر از تضاد خانواده با شغل بود که در ابعاد زمان و کشش نقشی قابل ملاحظه‌تر بود. همچنین این دو متغیر با هم ارتباط معنی‌دار، مستقیم و خطی داشتند (01/0 > P، 388/0 = r). از سوی دیگر، میزان کیفیت زندگی بیشتر پرستاران (90 درصد) در حد متوسط بود. کیفیت زندگی با دو متغیر تضاد شغل با خانواده (01/0 > P، 469/0- = r) و تضاد خانواده با شغل (05/0 > P، 282/0- = r) ارتباط معنی‌دار، معکوس و خطی داشت. نتیجه‌گیری: به نظر می‌رسد با توجه به وضعیت تضاد شغل پرستاری با خانواده و تأثیر آن بر کیفیت زندگی افراد شاغل، ارایه راهکارهای جامعی برای کاهش این تضاد در محیط کار اجتناب‌ناپذیر می‌باشد.

Title: اثربخشي برنامه آموزشي در ارتقای رفتارهاي پيشگيري كننده مصرف سيگار در نوجوانان

http://hsr.mui.ac.ir/index.php/jhsr/article/view/806

مقدمه: مصرف سيگار در نوجوانان يكي از مشكلات مهم بهداشتي محسوب مي‌شود و عامل خطر مهمي براي بروز رفتارهاي پرخطر در بزرگسالي است. با توجه به شروع مصرف سيگار در سنين پايين و خطرات ناشي از آن، توجه به سطح دانش و رفتارهاي پيشگيري كننده مصرف سيگار در نوجوانان ضروري مي‌باشد. بنابراین هدف از مطالعه حاضر، تعیین اثربخشی برنامه‌های آموزشی در ارتقای رفتارهای پیشگیری کننده مصرف سیگار در نوجوانان بود. روش‌ها: اين پژوهش يك مطالعه مداخله‌اي از نوع نيمه تجربي بود كه بر روي دانش‌آموزان پسر پايه دوم راهنمايي شهر شيراز در سال 89-1388 انجام گرفت. بر اين اساس و قبل از شروع مداخله، مطالعه‌اي در اين راستا انجام شد و طبق آن 240 نفر از دانش‌آموزان از دو مدرسه به صورت تصادفي، يكي به عنوان مداخله (120 نفر) و ديگري به عنوان شاهد (120 نفر) انتخاب شدند. سپس پرسش‌نامه در اختيار آنان قرار گرفت و پس از اجراي برنامه آموزشي در گروه مداخله، داده‌های لازم از دو گروه جمع‌آوري گردید و پس از ورود به نرم‌افزار SPSS نسخه 16 با استفاده از آزمون‌های آماری Independent t، Paired t، McNemar و 2c مورد تجزيه و تحليل قرار گرفت. يافته‌ها: ميانگين نمره آگاهي از عوارض و پيامدهاي مصرف سيگار بعد از مداخله آموزشي در گروه مداخله نسبت به گروه شاهد افزايش معني‌داري داشت (0001/0 > P). همچنين ميانگين نمره رفتارهاي پيشگيري كننده مصرف سيگار در گروه مداخله بعد از برنامه آموزشي افزايش معني‌دار آماري را نشان داد (0001/0 > P). نتيجه‌گيري: با توجه به نتایج مطالعه، بايد برنامه‌هاي آموزشي مناسبي در جهت تقويت رفتارهاي پيشگيري كننده مصرف سيگار براي پيشگيري و كنترل مصرف دخانيات در سنين پايين‌تر در نظر گرفته شود.

Title: بررسی میزان شیوع بیماری سیلیکوزیس و برخي عوامل مرتبط با آن در کارگران شاغل در کارگاه‌های (سنگ‌تراشی) شهرستان خمینی شهر

http://hsr.mui.ac.ir/index.php/jhsr/article/view/809

مقدمه: سیلیکوزیس (Silicosis) بیماری ریوی با سیر تکاملی آهسته و از بیماری‌های شغلی و غیر قابل برگشت می‌باشد که در مراحل ابتدایی فاقد علایم بالینی بوده و علامت اصلی آن تنها علایم رادیولوژیکی است. بروز این علایم بستگی به سن، حساسیت فردی، سابقه کار، مصرف سیگار و مدت زمان مواجهه دارد. هدف این مطالعه، بررسی شیوع بیماری سیلیکوزیس و عوامل مرتبط با آن در کارگران سنگ‌تراش خمینی شهری بود. روش‌ها: مطالعــه حاضر به صورت مقطعی، از طریق سرشماری روی70 سنگ‌تراش انجام گرفت. اطلاعات مورد نیاز از طریق معاینات کارگران (طبق فرم شماره 1 معاینات کارگران) و فرم ارجاع جمع‌آوری گردید. از تمام افراد گرافی قفسه سینه گرفته شد که با معاینه پزشک طب کار افراد مبتلا مشخص و دوباره از آن‌ها گرافی قفسه سینه با کیفیت بالا گرفته شد. تجزیه و تحلیل داده‌ها از طریق نرم‌افزار SPSS و به وسیله آزمون‌های 2c، Mann-Whitney و Independent t صورت پذیرفت. یافته‌ها: میانگین سن مبتلایان به سیلیکوزیس 04/18 ± 4/46 سال به دست آمد. 57/28 درصد افراد مبتلا کمتر از 20 سال سابقه کار داشتند، ضمن این‌که از 7 نفر مبتلا به این بیماری، 3 نفر سیگاری بودند. آزمون 2c رابطه معنی‌داری بین سیگاری بودن و ابتلا به سیلیکوزیس را نشان داد (05/0 = P). آزمونIndependent t رابطه معنی‌داری بین سابقه کار و سن افراد با سیلیکوزیس نشان نداد. نتیجه‌گیری: با توجه به ابتلای 6/15 درصد شاغلین، متولیان سلامت شغلی باید با برنامه‌ریزی صحیح‌تر در خصوص بهسازی شرایط محیط کار، معاینات به موقع پزشکی و کنترل عوامل زیان‌آور محیط کار نسبت به حفظ سلامت این افراد اقدام نمایند.

Title: ردیابی آنتروویروس‌ها در لجن دفعی نهایی (Biosolids)

http://hsr.mui.ac.ir/index.php/jhsr/article/view/811

مقدمه: میکروارگانیسم‌ها و پاتوژن‌های انسانی زیادی از جمله باکتری‌ها، ویروس‌ها و پروتوزئرها در لجن فاضلاب وجود دارند. بنابراین، لجن دفعی نهایی به عنوان یک آلاینده زیست محیطی و انتقال دهنده عوامل مسبب بیماری‌ها مورد توجه می‌باشد. آنتروویروس‌ها به عنوان عامل مبنایی گاستروانتریت‌های حاد و هپاتیت‌ها، یکی از مهم‌ترین پاتوژن‌های روده‌ای مؤثر بر سلامتی انسان هستند. هدف از این تحقیق، ردیابی آنتروویروس‌ها در لجن دفعی دو تصفیه‌خانه در اصفهان بود.  روش‌ها: در این تحقیق 30 نمونه از دو تصفیه‌خانه فاضلاب برداشت شد. بعد از اندازه‌گیری درجه حرارت، نمونه‌ها به آزمایشگاه منتقل و آزمایش‌ جامدات کل، جامدات فرار و آنتروویروس‌ها بر اساس استاندارد متد (بخش F 9510) و راهنمای کنترل پاتوژن و جذب ناقلین در لجن فاضلاب [EPA (Environmental Protection Agency) ضمیمه F] انجام شد. یافته‌ها: میانگین درجه حرارت نمونه‌ها 23 درجه سانتی‌گراد بود. میانگین تعداد آنتروویروس‌ها در دو تصفیه‌خانه A و B به ترتیب 106 ´ 5/4 و 105 ´ 7/7 واحد تشکیل دهنده پلاک (Plaque-forming unit یا PFU) در 4 گرم لجن دفعی نهایی بود. آنالیز آماری ارتباط معنی‌دار مهمی بین آنتروویروس‌ها و جامدات فرار نشان داد. تعداد آنتروویروس‌ها در تابستان در مقایسه با پاییز بیشتر بود. نتیجه‌گیری: نتایج نشان داد که نمونه‌ها نتوانستند الزامات کلاس A مرتبط با آنتروویروس‌ها را پوشش دهند و پتانسیل خطر برای مردم در مواجهه با لجن دفعی وجود دارد. بنابراین، محدودیت کاربرد در زمین برای حفاظت از سلامت عمومی و محیط زیست ضروری است. به هر حال برای کاهش پاتوژن‌ها و دستیابی به استانداردهای کاربرد لجن در زمین، باید روش‌های خاص تصفیه لجن مورد استفاده قرار گیرد.

Title: عوامل مرتبط با مصرف سیگار بر اساس سازه‌های مدل اعتقاد بهداشتی در دانش‌آموزان پسر مقطع پیش‌دانشگاهی شهر اصفهان

http://hsr.mui.ac.ir/index.php/jhsr/article/view/861

مقدمه: هدف از این مطالعه، تعیین ارتباط بین سازه‌های مدل اعتقاد بهداشتی (حساسیت درک شده، موانع درک شده، منافع درک شده، خودکارامدی درک شده و راهنما برای عمل) و مصرف سیگار در میان دانش‌آموزان پسر مقطع پیش‌دانشگاهی شهر اصفهان بود. روش‌ها: مطالعه توصیفی- تحلیلی حاضر به شیوه مقطعی بر روی 382 نفر از دانش‌آموزان پسر شهر اصفهان در سال 1390 انجام شد. در این مطالعه، آزمودنی‌ها از میان 8 مدرسه در سطح شهر اصفهان به شیوه تصادفی سیستماتیک انتخاب شدند. ابزار جمع‌آوری اطلاعات، پرسش‌نامه محقق ساخته بر اساس مدل اعتقاد بهداشتی بود. برای تحلیل داده‌ها از نرم‌افزار SPSS نسخه 18، آمار توصیفی و آزمون‌های 2c و Independent t استفاده شد. یافته‌ها: میانگین سنی دانش‌آموزان 62/0 ± 72/17 سال بود. در کل 2/7 درصد از آزمودنی‌ها در طول 30 روز گذشته سیگار کشیده بودند و 7/32 درصد از آن‌ها در طول زندگی خود تجربه مصرف سیگار را داشتند. نتایج آزمون t نشان داد که تفاوت آماری معنی‌داری بین حساسیت درک شده (03/0 = P)، منافع درک شده (01/0 = P)، خودکارامدی درک شده (001/0 = P) بین افراد سیگاری و غیر سیگاری وجود داشت. نتیجه‌گیری: نتایج این مطالعه نشان داد که بین سازه‌های مدل اعتقاد بهداشتی و مصرف سیگار در افراد مورد مطالعه ارتباط معنی‌دار آماری وجود دارد. بنابراین انتظار می‌رود توسعه برنامه‌های پیشگیری کننده از مصرف سیگار و یا برنامه‌های آموزشی با تمرکز بر سازه‌های مدل اعتقاد بهداشتی در این زمینه مفید واقع گردد.

Title: مقایسه تأثیر سه روش مداخله آموزشی بر اساس مدل باور بهداشتي بر انجام رفتار غربالگري ماموگرافي در زنان بالای 40 سال شهر اصفهان

http://hsr.mui.ac.ir/index.php/jhsr/article/view/868

مقدمه: سرطان پستان شایع‌ترین سرطان برای زنان در سراسر دنیا به شمار مي‌رود كه در صورت تشخيص در مراحل‌ اوليه، قابل كنترل می‌باشد. ماموگرافی دقیق‌ترین روش برای تشخیص زودرس سرطان پستان است. مطالعه حاضر به بررسی و مقایسه اثربخشی سه روش مختلف آموزشی بر ميزان آگاهی و باورهاي بهداشتي زنان بالای 40 سال شهر اصفهان نسبت به انجام رفتار غربالگري ماموگرافي پرداخت. روش‌ها: پژوهش حاضر از نوع مطالعه‌ای و تجربي بود كه به صورت مبتني بر جمعيت در میان 96 نفر از زنان بالاي 40 سال شهر اصفهان انجام شد. این زنان به طور تصادفی در 3 گروه مداخله آموزشی (آموزش گروهی همراه با کتاب، آموزش گروهی و ارسال پستی کتاب) قرار گرفتند. ابزار جمع‌آوری داده‌ها، پرسش‌نامه‌ای شامل سه بخش مشخصات فردی، سؤالات آگاهی و سؤالات مربوط به سازه‌هاي مدل اعتقاد بهداشتي (بر اساس پرسش‌نامه مدل اعتقاد بهداشتی Champion) می‌شد. داده‌های جمع‌آوری شده با استفاده از نرم‌افزار آماری SPSS نسخه 18 و از طریق آمار توصيفي و تحليلي 2c، ANOVA و Paired t در سطح معني‌داري 05/0 = α مورد تجزیه و تحلیل قرار گرفت. يافته‌ها: قبل از مداخله آموزشی اختلاف معني‌داري در ميانگين نمرات آگاهي و سازه‌هاي مدل اعتقاد بهداشتي بين سه گروه مورد مطالعه وجود نداشت؛ در حالي كه سه ماه بعد از مداخله اختلاف معني‌داري در ميانگين نمرات آگاهي و سازه‌هاي مدل بين سه گروه مورد مطالعه مشاهده شد. همچنين آزمون‌های آماري اختلاف معني‌داري بين ميانگين نمرات آگاهي و اغلب سازه‌های مدل اعتقاد بهداشتی در هر يك از گروه‌هاي مورد مطالعه، قبل و سه ماه بعد از مداخله نشان دادند (001/0 £ P). نتيجه‌گيري: در میان روش‌های مورد بررسی در این مطالعه، آموزش گروهی توأم با کتاب نسبت به دو روش دیگر تأثیر بیشتری بر افزایش آگاهی، حساسیت، شدت درک شده و منافع درک شده ماموگرافی و نیز کاهش موانع درک شده ماموگرافی داشت. این مسأله لزوم توجه به طراحی برنامه‌های آموزشی ترکیبی را می‌رساند.

Title: ارتقای مصرف میوه و سبزیجات در دانش‌آموزان مدارس ابتدايي شهر اصفهان

http://hsr.mui.ac.ir/index.php/jhsr/article/view/871

مقدمه: مطالعه حاضر با هدف بررسی اثربخشی مداخله آموزشی بر ارتقای مصرف میوه و سبزیجات در دانش‌آموزان پایه چهارم ابتدایی شهر اصفهان در سال 1389 انجام گرفت. روش‌ها: در این مطالعه تجربی، 138 نفر از دانش‌آموزان پایه چهارم ابتدایی به صورت تصادفی به دو گروه مداخله (68 نفر) و شاهد (70 نفر) تقسیم شدند. ابزار جمع‌آوری داده‌ها شامل پرسش‌نامه محقق ساخته و پرسش‌نامه ثبت غذايي بود. روایی و پایایی پرسش‌نامه به وسیله روایی صوری، روایی محتوا و پایایی درونی (86/0 = α) بررسی گردید. داده‌هاي هر دو گروه به صورت مشابه و در دو زمان قبل و 3 ماه پس از مداخله آموزشی جمع‌آوری شد. مداخله آموزش تغذیه در گروه آزمون در قالب 4 جلسه آموزشی 60 دقیقه‌ای و به صورت آموزش گروهی برگزار گردید. داده‌ها به وسیله آزمون‌هاي آماری 2c، Independent t و Paired t مورد تجزیه و تحلیل قرار گرفت. مشاركت افراد در اين تحقيق داوطلبانه و با رضايت آگاهانه بود. یافته‌ها: ميانگين نمره آگاهي (001/0 > P)، نگرش نسبت به رفتار (001/0 > P) و حمایت اجتماعی (030/0 = P) سه ماه پس از مداخله آموزشی در گروه مداخله بهبود معني‌داري يافت. علاوه بر آن، مقایسه رفتارهای تغذیه‌ای 3 ماه پس از مداخله آموزشی بهبود معنی‌داری را در میانگین سهم روزانه دریافت گروه غذایی میوه و سبزیجات در گروه مداخله نشان داد (001/0 > P). نتیجه‌گیری: نتایج مطالعه حاضر نشان می‌دهد که آموزش تغذیه به عنوان ابزاری در دسترس و مؤثر در برنامه‌های ارتقای سلامت با تمرکز بر توسعه رفتارهای تغذیه سالم قابل استفاده می‌باشد.

Title: بررسی ارتباط حمایت‌های اجتماعی با کیفیت زندگی بیماران مولتيپل اسکلروزيس در شهر اصفهان

http://hsr.mui.ac.ir/index.php/jhsr/article/view/913

مقدمه: يكي از بيماري‌هاي قرن حاضر كه متأسفانه اكثر جوان‌ها به خصوص دختران و زنان جوان به اين بيماري مبتلا مي‌شوند، بيماري مولتيپل اسکلروزيس (Multiple sclerosis یا MS) مي‌باشد. علت این بیماری هنوز از نظر پزشكان متخصص دنيا مشخص نيست. با توجه به تعداد زیاد این بیماران و اهمیت شناسایی عوامل مؤثر بر ارتقای کیفیت زندگی آنان، این مطالعه با هدف بررسی ارتباط بین حمایت‌های اجتماعی و کیفیت زندگی بیماران MS انجام گردید. روش‌ها: این مطالعه از نوع توصیفی با نمونه‌گیری تصادفی ساده در بین 230 نفر بیمار مبتلا به MS در شهر اصفهان (164 نفر زن و 66 نفر مرد) با ابزار پرسش‌نامه انجام گردید. متغیرهای مورد استفاده عبارت از حمایت‌های اجتماعی- اقتصادی، اجتماعی- فرهنگی، خدماتی، بهبودی نسب بیماران، رضایت از زندگی، ارتقای پایگاه اجتماعی بیمار، احساس امنیت، اعتماد به نفس، عبادت و افسردگی بودند. معیار سنجش متغیرها طیف لیکرت بود. پس از تعیین روایی و پایایی در مطالعه اولیه، داده‌های نهایی با سطح معنی‌داری 95 درصد و با استفاده از نرم‌افزار SPSS و آمارهای توصیفی و آزمون ضریب همبستگی Pearson تجزیه و تحلیل گردید.   یافته‌ها: در اين مطالعه 3/71 درصد پاسخگويان زن، 7/35 درصد متأهل و 9/30 درصد شاغل بودند. بين حمايت‌هاي اجتماعي با احساس رضايت از زندگي، ارتقای پايگاه و موقعيت فرد در اجتماع، اميد به زندگي، انگيزه جهت پذيرش علایم و اختلالات بيماري و افزايش اعتماد به نفس در بيماران MS (28/0 = r، 001/0 > P) رابطه معنی‌دار وجود داشت.   نتیجه‌گیری: نتایج حاکی از اهمیت حمایت‌های اجتماعی است که می‌تواند به احساس رضایتمندی از زندگی و امیدواری در بیماران مبتلا به MS منجر شود. حمايت‌هاي اجتماعي به صور گوناگون اعم از مالي- اقتصادي، فرهنگي- آموزشي و حمايتي- خدماتي مي‌تواند در رفاه حال بيماران مبتلا به MS مؤثر واقع گردد. با این وجود به سبب نبود درمان قطعي جهت درمان اين بيماري، حمايت‌هاي اجتماعي همه جانبه نيز نمي‌تواند با بهبودي نسبي اين مبتلايان رابطه معنی‌داري داشته باشد.

Title: اثربخشی برنامه آموزشی ارتقای آگاهی تغذیه‌ای در بیماران مبتلا به دیابت نوع دو با بهره‌گیری از مدل اعتقاد بهداشتی

http://hsr.mui.ac.ir/index.php/jhsr/article/view/958

مقدمه: آموزش تغذیه به بیماران مبتلا به دیابت برای کنترل بهتر بیماری یک ضرورت می‌باشد. هدف از انجام مطالعه حاضر تعیین اثربخشی برنامه آموزشی ارتقای آگاهی تغذیه‌ای روی بیماران مبتلا به دیابت نوع دو با بهره‌گیری از مدل اعتقاد بهداشتی بود. روش‌ها: این پژوهش یک مطالعه مداخله‌ای نیمه تجربی بود که روی بیماران مبتلا به دیابت نوع دو مراجعه‌کننده به مراکز بهداشتی درمانی روستایی شهرستان گچساران انجام شد. برای این امر از مسؤولین مرکز بهداشت شهرستان گچساران مجوز اخذ گردید و با مراکز بهداشتی درمانی روستایی هماهنگی صورت گرفت. سپس به تصادف بیماران 2 مرکز بهداشت به عنوان گروه مداخله و بیماران 2 مرکز دیگر به عنوان گروه شاهد در نظر گرفته شد. به تعداد 140 نفر از بیماران (70 نفر در گروه شاهد و 70 نفر در گروه مداخله) به صورت تصادفی ساده از بین بیماران جهت ورود به مطالعه انتخاب شدند. گروه مداخله آموزش‌های مورد نظر را دریافت نمودند. در نهایت پس از گذشت 3 ماه از انجام مداخله آموزشی از هر دو گروه ارزشیابی نهایی به عمل آمد. یافته‌ها: بعد از اجرای برنامه آموزشی اختلاف معنی‌داری در افزایش میانگین نمره آگاهی، شدت درک‌شده، حساسیت درک‌شده، منافع درک‌شده و خودکارامدی درک‌شده و کاهش موانع درک‌شده در بین بیماران گروه مداخله حاصل شد (05/0 > P). نتیجه‌گیری: نتایج مطالعه سودمندی اجرای برنامه‌های آموزشی در خصوص ارتقای آگاهی تغذیه‌ای در بین بیماران گروه مداخله را نشان داد.

Title: ارزیابی اثر تغییر برنامه نوبت کاری از نظام 12 ساعتی به نظام 8 ساعتی بر شیوع مشکلات بهداشتی در یک شرکت پتروشیمی

http://hsr.mui.ac.ir/index.php/jhsr/article/view/1043

مقدمه: مطالعه‌ای روی نظام نوبت‌کاری و مشکلات ناشی از آن در پرسنل نوبت‌کار یک شرکت پتروشیمی انجام شد. در آن مطالعه پیشنهاداتی برای تغییر نظام نوبت‌کاری ارایه گردید و آن شرکت نظام نوبت‌کاری خود را از نظام 12 ساعتی به نظام 8 ساعتی تغییر داد. پس از سپری شدن یکسال، این مطالعه با هدف ارزیابی اثر تغییر برنامه نوبت‌کاری بر میزان شیوع مشکلات بهداشی در ميان نوبت‌کاران شاغل در آن شرکت انجام شد. روش‌ها: نمونه‌ها در این مطالعه مقطعی شامل 79 نوبت‌کار مطالعه فعلی و 74 نوبت‌کار مطالعه قبلی بودند. ابزار جمع‌آوری داده‌ها مشابه مطالعه قبلی و متشکل از پرسش‌نامه‌ای بود که سؤال‌هایی در زمینه ويژگي‌هاي دموگرافيک، مشکلات بهداشتی و نظرخواهی در مورد تغییر برنامه نوبت‌کاری در آن گنجانده شده بود. تجزیه و تحلیل داده‌ها با استفاده از نرم‌افزار SPSS نسخه 5/11 انجام گرفت. یافته‌ها: میانگین سابقه‌کار در نظام نوبت‌کاری افراد مورد مطالعه در مطالعه فعلی و قبلی به ترتیب 40/2 ± 91/5 و 79/3 ± 18/5 سال بود (05/0 P >). شیوع برخی مشکلات گوارشی و اختلالات اسکلتی- عضلانی در مطالعه فعلی به طور معنی‌داری بیشتر از مطالعه قبلی بود (05/0 P <)، اما شیوع برخی مشکلات ذهنی در بین نوبت‌کاران مطالعه فعلی به طور معنی‌داری کمتر از شیوع آن‌ها در بین نوبت‌کاران مطالعه قبلی بود (05/0 P <). برنامه جدید از نظر نوبت‌کاران کمیت و کیفیت خواب آنان را بهبود بخشیده بود. همچنین یافته‌های این مطالعه نشان داد که 5/97 درصد افراد مورد مطالعه، نظام جدید را نسبت به نظام قبلی بهتر می‌دانستند. نتیجه‌گیری: بر اساس نتایج مطالعه حاضر، تغییر برنامه نوبت‌کاری از نظام 12 ساعتی به 8 ساعتی باعث کاهش شیوع اختلالات ذهنی در نوبت‌کاران شده بود و به بهبود وضعیت خواب و زندگی فردی، خانوادگی و اجتماعی انجامیده بود. بنابراین می‌توان چنین نتیجه‌گیری نمود که تغییر ایجاد شده در برنامه نوبت‌کاری مفید بوده است و اغلب افراد آن را اثربخش و مثبت ارزیابی نمودند.

Title: شیوع چاقی و اضافه وزن در دانشجویان دانشکده پزشکی

http://hsr.mui.ac.ir/index.php/jhsr/article/view/931

مقدمه: بررسی‌های اخیر حاکی از افزایش شیوع هشداردهنده چاقی در سراسر جهان می‌باشد. با توجه به افزایش آمار چاقی، اضافه وزن و چاقی شکمی در جامعه ایرانی و ارتباط این عارضه با شیوه زندگی و ساختارهای اجتماعی و شغلی، بررسی وضعیت شاخص‌های تن‌سنجی و میزان شیوع آن‌ها جهت انجام مطالعه‌ها و تحقیق‌های بعدی ضروری به نظر می‌رسد. هدف از انجام این تحقیق بررسی میزان شیوع چاقی، اضافه وزن و چاقی شکمی در دانشجویان دانشکده پزشکی دانشگاه علوم پزشکی اصفهان بود. روش‌ها: در این مطالعه توصیفی، 215 نفر دانشجوی شاغل به تحصیل در رشته‌ها و مقاطع مختلف دانشکده پزشکی دانشگاه اصفهان به طریق نمونه‌گیری تصادفی خوشه‌ای انتخاب شدند. علاوه بر تکمیل فرم اطلاعاتی مشخصات آنتروپومتریک آنان طبق پروتکل استاندارد اندازه‌گیری شد. یافته‌ها: در این مطالعه مشخص شد که صفر درصد دختران و 88/5 درصد پسران دچار اضافه وزن (kg/m2 25 > Body mass index یا BMI)، صفر درصد دختران و 92/3 درصد پسران دچار چاقی (kg/m2 30 BMI >) و 31/5 درصد دختران و 95/4 درصد پسران دارای چاقی شکمی بودند. درصد افراد بدون این عارضه در دو گروه دختران و پسران اختلاف کمی داشت (7/52 در برابر 3/47 درصد). این شیوع‌ها با افزایش سن و سابقه فامیلی نسبت مستقیم داشت. نتیجه‌گیری: در این مطالعه شیوع اضافه وزن، چاقی و چاقی شکمی تفاوت چندانی در گروه‌های مختلف سنی، جنسی و حتی بر اساس سابقه فامیلی نداشت. پیشنهاد می‌گردد که عوامل خطر بروز چاقی، اضافه وزن و چاقی شکمی و عوامل مؤثر بر آن در مطالعه‌های آینده مورد بررسی قرار گیرد.
